# Tooth Shade and Skin Colour: A Descriptive Cross-Sectional Study

**DOI:** 10.31729/jnma.4792

**Published:** 2020-03-31

**Authors:** Dilesh Pradhan, Lajana Shrestha, Junu Lohani

**Affiliations:** 1 Department of Prosthodontics, Kathmandu Medical College, Duwakot, Bhaktapur, Nepal

**Keywords:** *Nepal*, *skin colour*, *tooth shade*

## Abstract

**Introduction:**

Selection of proper tooth shade is one of the most significant factors influencing patients' aesthetic perception and improved prosthesis acceptance. Guidelines in the dental literature suggest age, sex, colour of skin, hair and eye for selecting tooth shade when past records cannot be obtained. The objective of the study was to find out the prevalence of the most common tooth shade in relation to the skin colour and the prevalence of the same in relation to age and sex.

**Methods:**

A descriptive cross-sectional study was carried out at Kathmandu Medical College from June to August 2019. Vitapan Classical Shade guide was used to select the shade of upper right central incisor in 338 participants. Revlon Foundation Makeup Shade guide was used to determine colour of skin. Participants were examined without facial makeup. Skin colour and teeth shade were examined in daylight at about the same time of the day. Data obtained were computed and analysed using Microsoft Excel 2016 software.

**Results:**

Prevalence of tooth shade with high value (lighter shade) was seen in all fair 121 (35.8%), medium 63 (18.6%) and dark skin tones 23 (6.8%). Most common teeth shade in fair individuals was B1 47 (37.9%), in medium also B1 25 (7.4%) and dark was B2 9 (2.7%). Age range of 10 to 35 years had tooth shade with higher value 159 (47.04%).

**Conclusions:**

Hence, teeth shade with high value (lighter shade) was prevalent in skin tone of all types in current study. Skin tone was not related to teeth shade selection, teeth became darker with age and females had lighter teeth shade (high value).

## INTRODUCTION

Denture aesthetics is defined as “the effect produced by a dental prosthesis that affects the beauty and attractiveness of the person”.^1^ A greater challenge to a dentist is to harmonise the surrounding environment (hair, skin, eye colour, and age) to enhance the overall facial aesthetics. Selection of proper tooth shade is one of the most significant factor influencing the patient's aesthetic perception and improved prosthesis acceptance.^2^

Factors suggested as guidelines for selection of tooth shade include age, sex, and colour of skin, hair, and eye.^3,4^ Some studies consider the skin tone to be more predictable reference for artificial tooth selection stating that individuals with darker skin colours have lighter shades of teeth while others found no relationship.^5,6^

The objective of this study was to find out the prevalenec of the most common tooth shade in relation to the skin colour, and to see the prevalence of the same in relation to age and sex.

## METHODS

This descriptive cross-sectional study was carried out at Kathmandu Medical College from June to August 2019. Ethical approval for the study was granted by Institutional Review Committee of Kathmandu Medical College on 20th May, 2019.

Convenient sampling method was used for data collection. Sample size was calculated using following formula:

n = Z^2^ × p × q/e^2^ = 251.41 ≈ 252;

p= prevalence, 38%^7^
q= 1-p
Z= 1.96, at 95% confidence interval
e= margin of error, 6%

Participants with fully erupted permanent upper central incisors without any endodontic therapy or other dental procedures that may alter the original shade of the teeth were selected. Students and individuals who visited the dental out-patient department were invited to participate in the study. Teeth with intrinsic or extrinsic stains and volunteers with diseases of the skin were excluded from the study. About 370 met the inclusion criteria. After explaining the nature and objective of the study 32 participants withdraw from the study and written consent was obtained from remaining 338 participants. Participants were divided into two groups: Group 1 included the individuals with lowest age to 35 years and Group 2 included individuals from 36 years to the highest age meeting the inclusion criteria.^8^

Vitapan Classical Shade guide was used to select the shade of the upper right central incisor. It consists total of 16 shade tabs. It was arranged it order of higher to lower value. Shades B1, A1, B2, D2 were categorised as high value, shades A2, C1, D2, D4, A3, D3, B3, A3.5 were categorised medium value and B4, C3, A4, C4 were categorised as low value shades. Manufacturer instructions and guidelines from the standard text books were followed to select the shade. Revlon Foundation Makeup Shade guide was used to determine the colour of the skin. It consists of eight shades, namely: vanilla, shell, nude, natural beige, medium beige, cool beige, golden beige, and rich ginger. The skin complexion of the participants was divided into three categories: fair, medium, and dark. Those with vanilla, shell, and nude were categorised as fair. Natural beige, medium beige and cool beige were categorised as medium and golden beige and rich ginger were categorised as dark complexion.

Participants were requested to remove their facial makeup if any before examination. Skin colour and teeth shade were examined and noted by a single examiner in day-light at about same time of the day for all the participants. The data obtained were computed and analysed using Microsoft Excel 2016 software.

## RESULTS

Of the total 338 participants, 130 (38.46 %) were male and 208 (61.54%) were females. Participants with all fair, medium, and dark skin complexion were found to have teeth shade with high value (Table 1),

**Table 1:** Relationship of skin colour with tooth value.

Skin	Value			Total
Colour	High value	Medium value	Low value	
Fair	121 (35.8)	58 (17.2)	5 (1.5)	184 (54.4)
Medium	63 (18.6)	46 (13.6)	1 (0.3)	110 (32.5)
Dark	3 (6.8)	19 (5.6)	2(0.6)	44 (13)

B1 being most common in fair and medium complexion skin, and B2 in dark skin (Table 2).

**Table 2:** Frequency of tooth shade in relation to skin colour.

Skin	Shade													
Colour	A1	A2	A3	A3.5	A4	B1	B2	B3	B4	C1	C2	C3	C4	D2
Fair	40 (11.8)	31 (9.2)	11 (3.3)	5 (1.5)	1 (0.3)	47 (13.9)	34 (10.1)	2 (0.6)	1 (0.3)	7 (2.1)	2 (0.6)	1 (0.3)	2(0.6) 0
Medium	24 (7.1)	20 (5.9)	10 (3.0)	3 (0.9)	1 (0.3)	25 (7.4)	12 (3.6)	7 (2.1)	0	2 (0.6)	4 (1.2)	0 0	2 (0.6)
Dark	7 (2.1)	5 (1.5)	6 (1.8)	2 (0.6)	2 (0.6)	7 (2.1)	9 (2.7)	2 (0.6)	0	3 (0.9)	1 (0.3)	0	0	0

The prevalence of teeth shade is presented in relevance to the age category (Table 3) and gender (Table 4).

**Table 3:** Age category and teeth shade value.

Age category	Value			Total
	High value	Medium Value	Low Value	
10 - 35	159 (47.04)	51 (15.08)	1 (0.29)	211 (62.43)
>35	48 (14.20)	72 (21.30)	7 (2.07)	127 (37.57)

**Table 4:** Frequency of tooth shade in relation to gender.

	Shade													
Sex	A1	A2	A3	A3.5	A4	B1	B2	B3	B4	C1	C2	C3	C4	D2
Male	25 (7.39)	25 (7.39)	9 (2.66)	6 (1.77)	3 (0.88)	22 (6.5)	22 (6.5)	7 (2.07)	5 (1.47)	5 (1.47)	-	-	1 (0.29)
Female	46 (13.6)	31 (9.17)	18 (5.32)	4 (1.18)	1 (0.29)	57 (16.86)	33 (9.76)	4 (1.18)	1 (0.29)	7 (2.07)	2 (0.59)	1 (0.29)	2 (0.59)	1 (0.29)
Total	71 (21)	56 (16.56)	27 (7.98)	10 (2.95)	4 (1.18)	79 (23.37)	55 (16.27)	11 (3.25)	1 (0.29)	12 (3.55)	7 (2.07)	1 (0.29)	2 (0.59)	2 (0.59)

## DISCUSSION

Selection of the teeth shade is very meticulous process and one requires a good understanding of different parameters of colour such as hue, chroma, value, and translucency. For that, standard procedures must be followed and necessary requirements must be met. It needs to blend with the face and harmonise with the patient's complexion. Hue describes the dominant colour of an object, value describes the lightness or darkness of the colour (hue), and chroma represents degree of saturation of particular hue.^1^ Therefore, the Vitapan classical shade tab was rearranged in order of value from higher to lower.

We grouped the age range from 10 to 35 years and above 35 years because the secondary dentin starts to form at the approximate age and there is also wearing off of enamel layer.9 The result of the present study showed that with increasing age (>35 years), the teeth tend to be darker. Other studies have also reported similar findings.^5,10,11^ As explained by Hartmann,^11^ the darkening of the dentin leads to an altered colour in aged teeth.

In relation to gender, it was observed that females had teeth shades with higher value (lighter teeth shades) compared to male. This finding is similar to previous study done by Dosumu and Dosumu.^6^ The reason could be females are more concerned about aesthetics than males and they have better oral hygiene practices.

In the current study, population with all fair, medium, and dark skin tone were observed to have teeth shade with high value, B1 being the most common shade in fair and medium group, and B2 in dark skin. The reason may be due to the age group of the study sample, which is 62% below 35 years, in whom secondary dentin is yet to form. Like earlier studies,^5,10,12^ the result didn't find any relation between skin tone and teeth colour. This is in contrary to the findings of Sharma et al.^13^ and Vadavadagi et al.^7^ who reported positive association between the skin tone and tooth shade. However, the prevalence of teeth with lighter shades for medium and dark skin tone matched with findings from current study.

The limitation of this study is that the data was extracted from a specific population, that is only individuals visiting the dental out-patient department were recruited. The result would have been more informative if data extraction was done from diverse population.

## CONCLUSIONS

All skin tones with fair, medium, and dark complexion were found to have shades with high value (lighter shades) and is not related to the selection of the teeth shade. Teeth tend to get slightly darker with advancing age. In current study population, females had lighter teeth shades (high value) compared to male.
